# Circulating fibrocyte percentage and neutrophil-lymphocyte ratio are accurate biomarkers of uncomplicated and complicated appendicitis: a prospective cohort study

**DOI:** 10.1097/JS9.0000000000000234

**Published:** 2023-02-16

**Authors:** Mohamed Zarog, Peter O’Leary, Miranda Kiernan, Jarlath Bolger, Paul Tibbitts, Stephen Coffey, Gerard Byrnes, Colin Peirce, Colum Dunne, Calvin Coffey

**Affiliations:** a Department of Surgery, University Hospital Limerick; b School of Medicine; c Centre for Interventions in Infection, Inflammation and Immunity (4i), University of Limerick, Limerick, Ireland

**Keywords:** biomarker, complicated appendicitis, fibrocytes, uncomplicated appendicitis

## Abstract

There is increasing evidence that uncomplicated appendicitis (UA) may be treated nonoperatively in cases of UA. This study aimed to evaluate and compare the diagnostic accuracy of circulating fibrocyte percentage (CFP), white blood cell count, C-reactive protein, and neutrophil-lymphocyte ratio (NLR) in diagnosing uncomplicated and complicated appendicitis.

**Materials and methods:**

Eighty consecutive adult patients presenting with suspected appendicitis were recruited in a cohort-based prospective study between June 2015 and February 2016 at University Hospital Limerick in Ireland. Peripheral venous samples were obtained at the presentation. Clinical, biochemical, radiological, and histopathological parameters were recorded. The CFP was determined by dual-staining for CD45 and collagen-I using flow cytometry analysis and correlated with histopathological diagnoses.

**Results:**

Of the 46 patients who underwent appendicectomy, 34 (73.9%) had histologically proven acute appendicitis. A comparison of the diagnostic accuracy of biomarkers demonstrated the CFP had the highest diagnostic accuracy for UA (area under the curve*=*0.83, sensitivity*=*72.7%, specificity*=*83.3%, *P=*0.002). The NLR had the highest diagnostic accuracy in relation to complicated appendicitis (area under the curve*=*0.84, sensitivity*=*75.5%, specificity*=*83.3%, *P=*0.005).

**Conclusions:**

CFP and NLR are accurate biomarkers of UA and complicated appendicitis.

Highlights

- Patients with uncomplicated appendicitis could be managed nonoperatively.
- The circulating fibrocyte percentage is increased in patients with uncomplicated appendicitis.
- The neutrophil-lymphocyte ratio outperformed other biomarkers in diagnosing patients with complicated appendicitis.

## Background

Acute appendicitis is the most common cause of emergency surgical intervention^1^. The diagnosis of acute appendicitis can be challenging^2^,^3^. Early recognition of the severity of acute appendicitis is important, as this influences whether emergency surgical intervention is required.

Laparoscopic appendicectomy is considered the gold standard treatment for acute appendicitis^4^–^6^. Albert Ochsner first described the nonoperative approach in treating acute appendicitis with antibiotics only in select patients^7^. Coldrey reported that he conservatively treated 137 patients with acute appendicitis, with low mortality (0.2%) and recurrence rates (14.4%)^8^.

Uncomplicated appendicitis (UA) is defined as appendicitis without perforation, peritonitis, abscess, or coprolite^9^. Complicated appendicitis is inflammation of the appendix with secondary pathology (gangrenous, perforation, abscess, or peritonitis)^10^. Recent studies have suggested that UA may be managed nonoperatively with antibiotics only^6^,^11^,^12^. In high-risk groups conservative management of UA may be preferable^13^–^15^.

Infection with the novel coronavirus disease 2019 (COVID-19) is associated with high rates of postoperative morbidity and mortality^16^. In keeping with this, the nonoperative approach may become preferable in patients diagnosed with acute appendicitis in the COVID-19 era^17^,^18^. However, the nonoperative approach is associated with up to 40% risk of recurrence of appendicitis in the first year of follow-up^11^,^19^. In addition, appendiceal inflammation may progress to perforation or periappendiceal abscess/phlegmon formation. These are associated with high levels of morbidity and possibly even mortality^20^–^22^. Given the above, there is a need to identify patients with UA who might subsequently be managed nonoperatively.

A number of modalities help in differentiating between uncomplicated and complicated appendicitis. Computerized tomographic imaging remains the most sensitive and specific test^23^,^24^. However, radiation exposure, availability, and cost-effectiveness are the main limitations of this modality^25^,^26^. C-reactive protein (CRP) is a biomarker commonly used to determine the risk of perforation of the appendix. More recently, the neutrophil-lymphocyte ratio (NLR) has emerged as a biomarker for complicated appendicitis^27^,^28^.

Circulating fibrocytes (CFs) are hematopoietic cells derived from the bone marrow^29^. They secrete cytokines and collagen-1^30^. They promote tissue healing and thus contribute to scar formation. Previous studies demonstrated an association between CFs and inflammatory diseases^31^,^32^. CFs are involved in fibroinflammatory intestinal conditions such as Crohn’s disease and mesenteric panniculitis^33^–^35^. CFs contribute to the pathogenesis of respiratory diseases such as asthma, chronic obstructive pulmonary disease, and idiopathic pulmonary fibrosis^31^,^36^,^37^. Previous work by our group has shown that the circulating fibrocyte percentage (CFP) is elevated in patients with acute appendicitis^38^–^42^.

Given the above, the aim of this study was to identify biomarkers that could differentiate between UA and a normal appendix and complicated appendicitis and a normal appendix in patients presenting with suspected appendicitis.

## Materials and methods

Ethical approval was obtained from the Research Ethics Committee at University Hospital Limerick (record number 109/15). The study was registered at https://clinicaltrials.gov/ct2/show/NCT03988660. This study has been reported in line with the STROCSS 2019 Guideline checklist^43^.

### Study design

**Figure 1:**
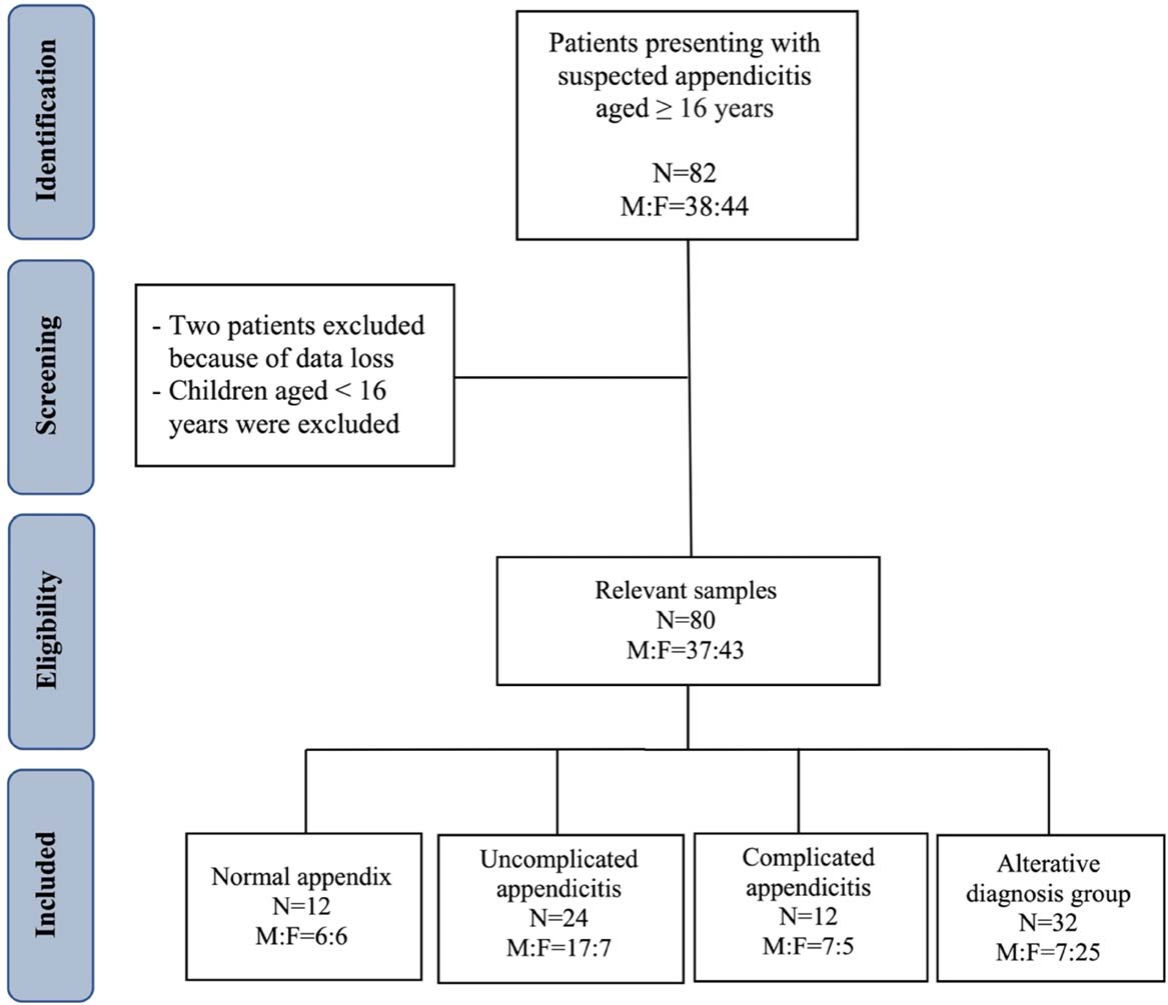
Flow chart of study selection process. F, female; M, male.

This prospective cohort study was performed in patients (age ≥16 y) presenting with suspected appendicitis at University Hospital Limerick in Ireland between June 2015 and February 2016. Patients under 16 years old were excluded from the study, as they have different cellular and hematologic profiles (Fig. 1). A peripheral venous blood sample was taken after obtaining informed consent. Collected data included each patient’s sex, age, presenting symptoms, duration of symptoms, white blood cell count (WCC), CFP, NLR, CRP, diagnosis on discharge, the operation performed, and postoperative histological analysis results.

### Determination of the proportion of fibrocytes in circulating monocytes

Samples were processed as previously described by Coffey *et al*.^33^. A single 10 ml sample of heparinized venous blood was collected via peripheral or upper extremity venipuncture. Samples were collected in sodium heparin (EDTA) vacutainer tubes and transferred to the laboratory at the University of Limerick. Samples were processed to isolate the buffy coat using density gradient centrifugation (Histopaque; Sigma-Aldrich). The resulting peripheral blood mononuclear cells were subsequently washed in PBS and re-suspended in freezing medium (50% fetal bovine serum, 40% Roswell Park Memorial Institute medium, and 10% dimethyl sulfoxide) prior to transfer to cryogenic vials in 1 ml aliquots. Finally, samples were cooled in a cryogenic temperature control rate container to −80°C until processing for flow cytometry.

### Flow cytometry analysis

**Figure 2:**
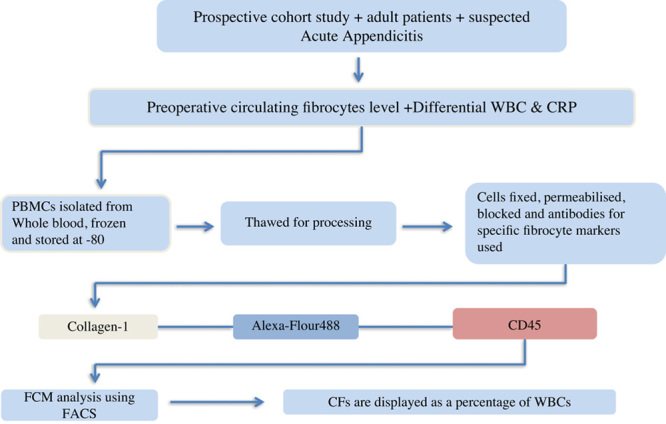
Flow chart shows process of identification of circulating fibrocyte percentage. CD, cluster of differentiation; FCM, flow cytometry analysis; FACS, fluorescence-activated cell sorting; WBC, white blood cells.

Flow cytometry analysis was performed as described previously in Coffey *et al*.^33^. Following white blood cell isolation using density gradient centrifugation, 1×10^6^ cells were re-suspended in flow cytometry analysis buffer (Roswell Park Memorial Institute medium supplemented with 10% horse serum, 0.1% sodium azide, and 25 mmol/l HEPES). Cells were fixed and permeabilized using BD Cytofix/Cytoperm solution (BD Biosciences) and blocked prior to intracellular staining of collagen-I with mouse antihuman collagen-I antibody (Product code MAB3391; Millipore). These were then stained with Alexa-Fluor 488 goat antimouse secondary antibody (Product code 115-545-146; Jackson ImmunoResearch Europe). Cells were finally stained for cell surface antigen CD45 using PerCP antihuman CD45 (Biolegend), re-suspended in PBS, and analyzed on the flow cytometer (BD FACSVerse)^44^–^46^. All analysis was done on a BD FACSVerse (BD Biosciences) using BD FACSuite v1.0.5 (BD Biosciences). CF levels were displayed as a percentage of the total white blood cell population (Fig. 2).

### Patient cohort

Patients were categorized into three cohorts based on preoperative radiological imaging, intraoperative macroscopic findings, and postoperative histological analysis. These cohorts were the normal appendix group, the UA (simple or edematous) group, and the complicated appendicitis (necrotic, gangrenous, perforated, mass, or peritonitis) group. The normal appendix cohort was adopted as a reference cohort.

### Statistical analysis

Data analyses were performed using IBM SPSS for Mac OSX Version 25.0. The distribution of variables was assessed by histograms and box plots. The continuous variables with a normal distribution were presented as the mean and SD and those without a normal distribution were presented as medians and interquartile ranges. Mann–Whitney *U*-test was utilized for nonparametric comparisons. *χ* ^2^-test was used to compare nominal variables. Multivariable analysis was used to compare the biomarkers in UA and the normal appendix cohorts, as well as complicated appendicitis and the normal appendix cohorts. Receiver operating characteristic curves were used to characterize and compare the diagnostic accuracy of the biomarkers. The Pearson test was used to determine correlations between the biomarkers. A two-tailed *P* value of less than 0.05 was considered statistically significant.

### Missing data

Two of the 82 (2.4%) patients missed different data during the analysis. These were not included in the final analysis.

## Results

### Demographics

Eighty patients with suspected acute appendicitis were recruited at University Hospital Limerick in the period between June 2015 and February 2016. Forty-eight of 80 (60%) patients had a final diagnosis of acute appendicitis based on clinical parameters. Forty-six of the 48 (95.8%) patients with clinically confirmed acute appendicitis were treated surgically with laparoscopic appendicectomy (with no conversion to open surgery). Of the 46 patients who underwent laparoscopic appendicectomy, 12 patients had a normal appendix on postoperative histological analysis (a negative appendicectomy rate of 26%). Males and females were equally distributed in the normal appendix cohort. The median age of patients in the normal appendix cohort was 25 years (Table 1).

**Table 1:** Basic characteristics

	Normal appendix (*n*=12)	Uncomplicated appendicitis (*n*=24)	Complicated appendicitis (*n*=12)	Alternative diagnosis (*n*=32)
Sex				
Male	6	17	7	7
Female	6	7	5	25
Age (years)	25.5±32.0	25.0±17.0	31.5±18.0	38.5±23.0

Of those who underwent surgery, 24 (30%) patients had UA and were included in the UA cohort. There were more males in the UA cohort (*P=*0.041). The median age of patients in the UA cohort was 25 years (Table 1).

Ten patients had complicated appendicitis on postoperative histological analysis and were thus included in the complicated appendicitis cohort. A further two patients had complicated appendicitis based on radiological imaging and were included in the complicated appendicitis cohort. Overall, 15% of patients presenting with suspected acute appendicitis had a final diagnosis of complicated appendicitis (*n=*12). The median age of patients in the complicated appendicitis cohort was 31 years (Table 1).

Of the 80 patients with suspected appendicitis, 32 (40.0%) had a final diagnosis other than appendicitis. Of these, 16 (50.0%) were diagnosed with nonspecific abdominal pain, and four (12.5%) had ovarian pathology (Table 2).

**Table 2:** Alternative diagnosis group

Alternative diagnosis group	Number
Nonspecific abdominal pain	16
Ovarian pathology	4
Diverticulitis	2
Terminal ileitis	1
Uterine fibroids	1
Ureteric stone	1
Constipation	1
Urinary tract infection	1
Mesenteric adenitis	1
Acute cholecystitis	1
Adhesions	1
Colonic tumor	1
Subcutaneous hematoma	1
Total	32

### Characterization of biomarker levels in each cohort

**Figure 3:**
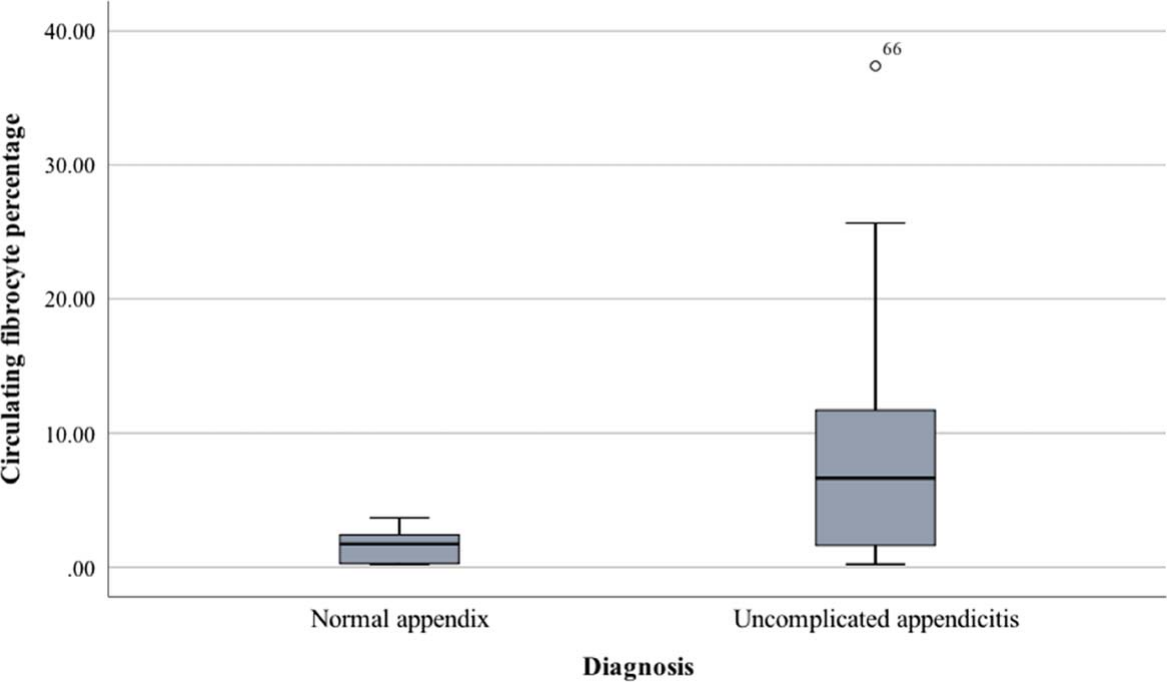
A box plot summarizing circulating fibrocyte percentage in histologically proven normal appendix and uncomplicated appendicitis.

Median levels of all biomarkers were increased in the UA cohort when compared with the normal cohort (Table 3, Figs. 2 and 3). Median levels of the WCC, CRP and NLR (but not the CFP) were increased in the complicated appendicitis cohort when compared with the normal cohort (Table 4).

**Table 3:** Demographic characteristics and biomarker values of histologically confirmed normal and uncomplicated appendicitis

Biomarker	Normal appendix	Uncomplicated appendicitis	*P* value
CFP	1.7±2.4	6.6±10.3	0.003
CRP (mg/l)	4.0±6.0	15.5±27.0	0.011
WCC (10^6^/μl)	7.0±4.6	11.1±6.3	0.032
NLR (10^6^/μl)	2.2±2.7	5.8±11.3	0.021

CFP, circulating fibrocyte percentage; CRP, C-reactive protein; NLR, neutrophil-lymphocyte ratio; WCC, white blood cell count.

**Table 4:** Demographic characteristics and biomarker values of histologically confirmed normal appendix and complicated appendicitis groups

Biomarker	Normal appendix	Complicated appendicitis	*P* value
CFP	1.7±2.4	2.9±10.2	0.114
CRP (mg/l)	4.0±6.0	37.5±126.0	0.006
WCC (10^6^/μl)	7.0±4.6	14.4±6.3	0.010
NLR (10^6^/μl)	2.2±2.7	8.6±8.0	0.004

CFP, circulating fibrocyte percentage; CRP, C-reactive protein; NLR, neutrophil-lymphocyte ratio; WCC, white blood cell count.

### Characterization of the relationship between biomarker levels

**Figure 4:**
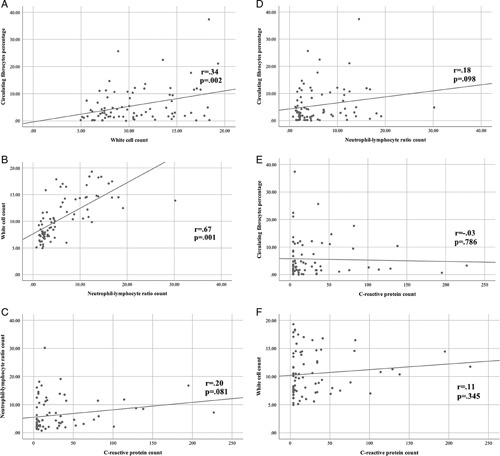
Correlations between biomarkers in patients presenting with suspected acute appendicitis.

The relationship between CFP and other biomarkers was assessed in patients presenting with suspected acute appendicitis. A direct positive correlation occurred between CFP and the WCC, and also between the WCC and NLR (*r*=0.34, *P=*0.002; *r*=0.67, *P=*0.001, respectively) (Fig. 4). No other correlations were apparent.

### Characterization of the diagnostic accuracy of biomarkers

**Figure 5:**
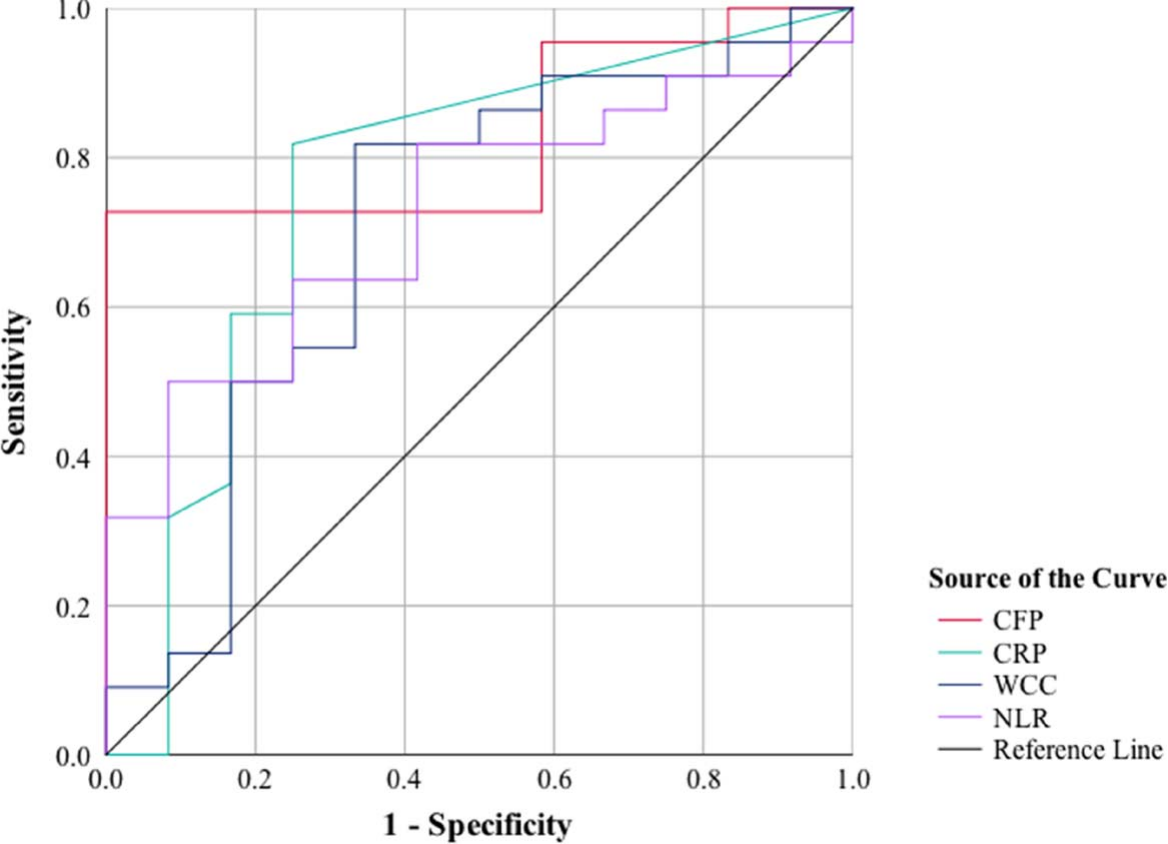
Receiver operator characteristic (ROC) curve showing the diagnostic accuracy of biomarkers [circulating fibrocyte percentage (CFP), C-reactive protein (CRP), white cell count (WCC), and neutrophil-lymphocyte ratio (NLR)] in uncomplicated appendicitis.

**Figure 6:**
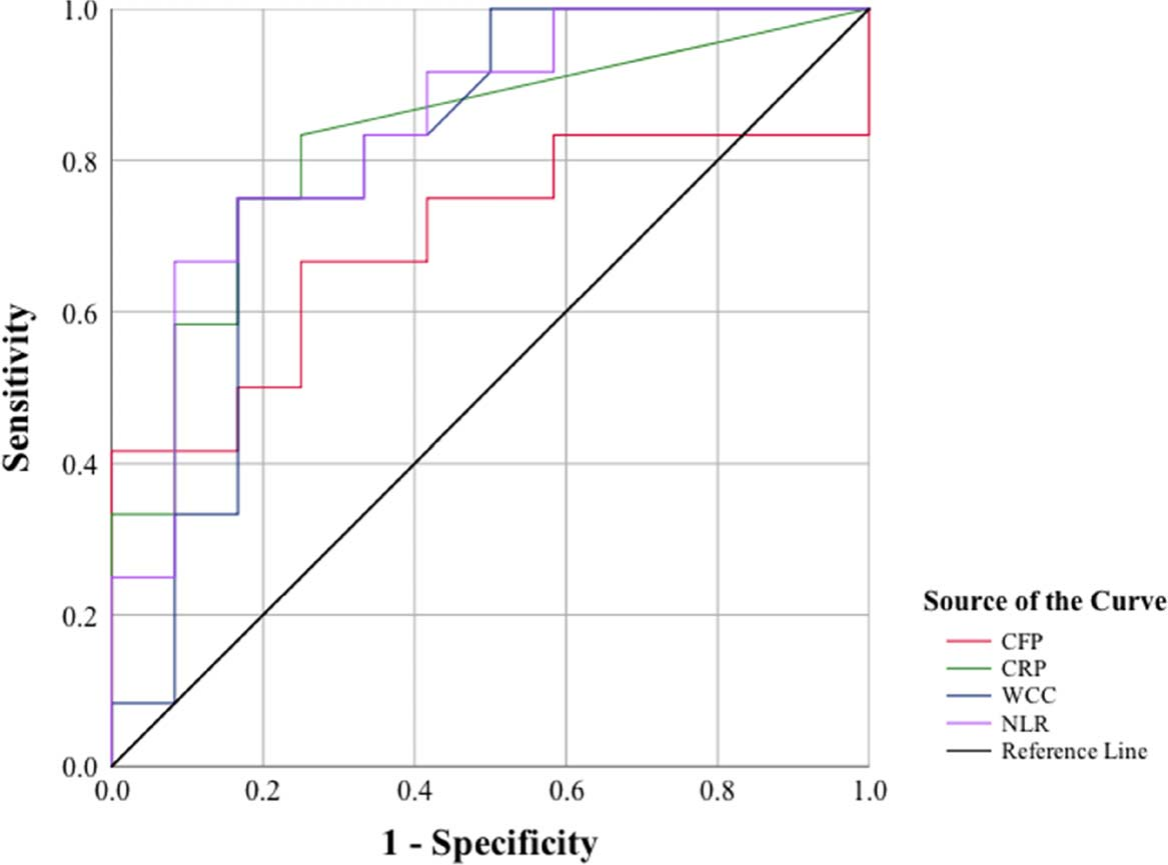
Receiver operator characteristic (ROC) curve showing the diagnostic accuracy of biomarkers [circulating fibrocyte percentage (CFP), C-reactive protein (CRP), white cell count (WCC), and neutrophil-lymphocyte ratio (NLR)] in complicated appendicitis.

The CFP had the highest area under the curve (AUC) in regards to the identification of UA (AUC=0.83, *P=*0.002; Fig. 5). A threshold CFP value of 3.2% had the highest sensitivity and specificity in terms of identifying UA (sensitivity and specificity of 72.7 and 83.3%, respectively) (Table 5). The NLR had the highest AUC in regards to the identification of complicated appendicitis (AUC=0.84, *P=*0.005; Fig. 6). A threshold NLR value of 5.6×10^6^/μl had the highest sensitivity and specificity in terms of identifying complicated appendicitis (sensitivity and specificity of 75.5 and 83.3%, respectively) (Table 6).

**Table 5:** Comparison between the diagnostic accuracy of biomarkers in uncomplicated appendicitis

The markers	AUC (95% CI)	Threshold value	Sensitivity (95% CI)	Specificity (95% CI)	PPV (95% CI)	NPV (95% CI)	*P*
CFP	0.83 (0.69–0.96)	3.2	72.7 (49.7–89.2)	83.3 (51.5–97.9)	88.8 (68.7–96.6)	62.5 (44.6–77.5)	0.002
CRP (mg/l)	0.75 (0.57–0.948)	8.0	68.2 (45.1–86.1)	75.0 (42.8–94.5)	83.3 (64.3–93.3)	56.3 (39.1–72.0)	0.014
WCC (10^6^/μl)	0.70 (0.50–0.90)	10.7	50.0 (28.2–71.9)	75.0 (42.8–94.5)	78.6 (55.8–91.4)	45.00 (32.5–58.2)	0.052
NLR (10^6^/μl)	0.72 (0.55–0.89)	4.4	54.5 (32.2–75.6)	75.0 (42.8–94.5)	80.0 (58.3–91.9)	47.4 (33.9–61.2)	0.033

AUC, area under the curve; CFP, circulating fibrocyte percentage; CRP, C-reactive protein; NLR, neutrophil-lymphocyte ratio; NPV, negative predictive value; PPV, positive predictive value; WCC, white blood cell count.

**Table 6:** Comparison between the diagnostic accuracy of biomarkers in complicated appendicitis

The markers	AUC (95% CI)	Threshold Value	Sensitivity (95% CI)	Specificity (95% CI)	PPV (95% CI)	NPV (%95 CI)	*P*
CFP	0.69 (0.46–0.92)	3.3	41.7 (15.8–72.3)	83.3 (51.6–97.9)	71.4 (37.4–91.3)	58.8 (45.4–71.0)	0.106
CRP (mg/l)	0.82 (0.65–0.99)	22.5	58.3 (27.7–84.8)	83.3 (51.6–97.9)	77.8 (47.5–93.1)	66.7 (49.4–80.4)	0.007
WCC (10^6^/μl)	0.80 (0.61–0.98)	10.6	75.5 (42.8–94.5)	75.0 (42.8–94.5)	75.0 (51.6–89.3)	75.0 (51.6–89.3)	0.012
NLR (10^6^/μl)	0.84 (0.67–1.00)	5.6	75.5 (42.8–94.5)	83.3 (51.6–97.9)	81.8 (54.9–94.3)	76.9 (54.8–90.2)	0.005

AUC, area under the curve; CFP, circulating fibrocyte percentage; CRP, C-reactive protein; NLR, neutrophil-lymphocyte ratio; NPV, negative predictive value; PPV, positive predictive value; WCC, white blood cell count.

## Discussion

Recent evidence suggests that UA may be treated conservatively^9^,^47^,^48^. However, almost all cases of complicated appendicitis require surgical intervention^49^,^50^. Given the above, it is important to distinguish uncomplicated and complicated appendicitis from a normal appendix. This study found that the CFP was an accurate biomarker of UA. The NLR was an accurate biomarker of complicated appendicitis. The findings indicate these biomarkers may be used in differentiating patients with uncomplicated and complicated appendicitis, and thus in helping select patients for different treatment pathways.

Delayed diagnosis of complicated appendicitis is associated with significant morbidity and mortality^51^. Patients with complicated appendicitis should be prioritized for emergency surgery. In this study, 42.5% of patients presenting with suspected acute appendicitis had histologically confirmed acute appendicitis. Of this cohort, 29.4% of patients had complicated appendicitis. 70.6% had UA. These were significant proportions of the overall cohort of patients presenting with suspected acute appendicitis and emphasis the importance of being able to differentiate complicated from uncomplicated cases.

In this study, 57% of patients presenting with suspected acute appendicitis underwent laparoscopic appendicectomy. Of this cohort, 26% had a histologically normal appendix. This was a noteworthy proportion of the overall cohort of patients who had laparoscopic appendicectomy. Negative laparoscopy appendicectomy may result in significant morbidity and even possibly mortality^52^. The data indicate that the CFP can differentiate patients with UA from those with a normal appendix. Similarly, the NLR may differentiate patients with complicated appendicitis from those with a normal appendix.

The above findings mean that, the CFP and NLR could be used to firstly differentiate patients with appendicitis from those with a normal appendix. Following that, these biomarkers could then be used to divide those with appendicitis into uncomplicated and complicated cohorts. Given patients with UA may sometimes be managed nonoperatively, the CFP may be used to identify those patients who may be managed conservatively. As complicated appendicitis normally requires operative intervention, the NLR could be used to select these patients from those with appendicitis. Operative intervention could then be instituted in that cohort.

The COVID-19 and other pandemics brought the debate between conservative versus surgical management of appendicitis back into the spotlight^53^. The risks associated with severe acute respiratory syndrome coronavirus-2 infection appear considerable and early on during the global pandemic, these mandated that nonoperative approaches to disease management be instituted where possible^16^,^17^. In keeping with this, it is important to develop means of identifying patients with uncomplicated or complicated appendicitis as those with UA are more likely to improve clinically with conservative management, compared to those with complicated appendicitis. Our results show that the CFP was an accurate biomarker of UA, and may serve as a means of identifying patients who may be suitable for conservative management. Our results also demonstrate that the NLR was an accurate biomarker of complicated appendicitis (i.e. abscess or perforation), and may serve as a means of identifying patients who may require direct intervention.

The present study was limited in that it was a single-center study in which patients aged less than 16 years were excluded. It compared relatively small-sized prospective cohorts. The findings demonstrate that trials aiming to further investigate the suggestion that serological biomarkers may be used to identify patients with uncomplicated or complicated appendicitis are increasingly required. A multicenter clinical trial with larger cohorts is warranted. Future studies could include a descriptive cost analysis of operative versus nonoperative treatment in UA. A further study could determine the economic impact of operative management of complicated appendicitis.

## Conclusion

The CFP was increased in UA and may differentiate between UA, and a normal appendix, in patients who present with suspected appendicitis. The NLR outperformed other biomarkers in differentiating complicated appendicitis and could represent a biomarker that differentiates complicated appendicitis, from a normal appendix, in patients who present with suspected appendicitis.

## Sources of funding

This work was supported by School of Medicine (University of Limerick) Strategic Research Fund.

## Ethical approval

Ethical approval was obtained from the Research Ethics Committee at University Hospital Limerick (record number 109/15). All patients have consented to participate on the study.

## Authors’ contributions

M.Z. collected and processed blood samples, clinical data collection, and designed the study, data analysis, and writing of manuscript. P.O. collected data, involved in study design and drafting of manuscript. M.K., P.T., S.C.: collected, processed blood samples. J.B. involved in study design, and clinical data collection. A.L., G.B., C.P., C.D. involved in study design and drafting of manuscript. J.C. involved in study design, overall supervision, and writing of manuscript. All authors read and approved the final manuscript.

## Conflicts of interest disclosure

The authors have no conflict of interest to declare.

## Research registration unique identifying number (UIN)

1. Name of the registry: ClinicalTrials.gov.

2. Unique Identifying number or registration: NCT03988660.

3. Hyperlink to your specific registration: https://clinicaltrials.gov/ct2/show/NCT03988660

## Guarantor

Mohamed Zarog and Calvin Coffey.

## Provenance and peer review

Not commissioned, externally peer-reviewed.

## Data statement

Please contact the authors for data requests.
